# A qualitative study exploring patient and healthcare practitioner perspectives about at-home fetal Doppler devices

**DOI:** 10.1038/s44294-026-00139-6

**Published:** 2026-04-17

**Authors:** Sabrina Keating, Rosa Mackay, Jennifer Maclellan, Abigail McNiven, Sharon Dixon

**Affiliations:** 1 Nuffield Department of Primary Care Health Sciences, University of Oxford, Oxford, UK; 2 Department of Population Health, National Perinatal Epidemiology Unit, University of Oxford, Oxford, UK

**Keywords:** Health services, Health care

## Abstract

At-home foetal Dopplers are widely sold for use outside of medical supervision, despite safety concerns regarding the potential for false-reassurance leading to delayed care-seeking. To learn more about pregnant peoples’ decision-making surrounding using these devices, we conducted semi-structured interviews with 20 women who had considered or used a home Doppler and interviews or focus groups with 15 healthcare practitioners (HCPs) (10 midwives, 1 obstetrician, 4 GPs). We conducted thematic analysis using a mind-mapping approach. Home Doppler users sought early reassurance and bonding, often before perceiving foetal movements. Although the devices were adopted to alleviate anxiety, they could paradoxically exacerbate it. HCPs worried about the potential for false reassurance, misinterpretation, and unregulated consumer devices. In healthcare encounters, both groups tended to avoid discussing home Doppler use, reducing opportunities to explore concerns and offer evidence-based interventions. Care should enable non-judgemental conversations about Dopplers while reiterating that they must not replace timely care-seeking.

## Introduction

At-home ‘foetal Dopplers’ (referred to herein as home Dopplers when discussion pertains to at-home, unsupervised use of a foetal Doppler) are handheld devices that use ultrasound waves to detect the movement of blood in vessels, producing amplified output through a built-in speaker^1^. The devices are marketed as allowing pregnant women to listen to their unborn baby’s heartbeat at home and at any time^2^. Prices vary widely, ranging from under ten to hundreds of pounds. While the devices are widely available and advertisers claim, or otherwise imply, that the devices are easy and safe to use, concerns have been raised^2^ and statements and guidance have been issued discouraging their use^1,3–6^.

Supervised home foetal heart rate monitoring has been used in specialist programmes for pregnancies complicated by foetal supraventricular tachyarrhythmia (SVT) or maternal anti-Ro/SSA antibodies^7–10^. In these programmes, women typically check the foetal heart rate twice daily and contact the clinical team if the rate is markedly elevated (e.g. >180 beats per minute), low (e.g. <100 beats per minute), or if they are unable to detect a rate. The team then provides prompt advice, which may include medication adjustment or urgent clinical assessment. However, safety concerns about unsupervised home foetal heart monitoring have been raised, including warnings for caution published in the *BMJ* in 2009 based on two pregnant women who noticed reduced foetal movements but were falsely reassured by home Doppler use and delayed accessing care^2,11^. In one case, the pregnancy ended in stillbirth. These articles caution against the use of home Dopplers, citing the risk of false reassurance from the maternal heartbeat or misinterpreting placental flow for the foetal heartbeat. Chakladar also raised the issue that increased uptake of home Dopplers would result in unnecessary care-seeking following inexperienced users being unable to find the foetal heart rate^2^. Following publication and awareness of cases such as these, medical bodies and advocacy organisations published guidance and ran campaigns which discourage the use of unsupervised home Dopplers^1,3–6^. A 2017 bill attempted to restrict the sale and use of the devices in the UK, but was never passed into law, with the devices remaining available for purchase^12^.

Against this backdrop of efforts to discourage or restrict the use of home Dopplers, there is some qualitative research exploring what users experience as potential benefits of the devices, including for pregnant women navigating anxiety around miscarriage^13,14^. Middlemiss conducted an interview-based study with home Doppler users in Cornwall (*N* = 17), and suggested that guidance simply warning against all use of home Dopplers overlooks the realities of pregnant women’s experiences and overgeneralises the risks of use^14^. Middlemiss’s study reported that home Dopplers were typically used to verify the presence of the baby’s heartbeat and to mitigate day-to-day anxiety, with this being desirable to those with previous experience of miscarriage or high-risk pregnancies. This study found that the devices were typically used prior to consistent foetal movements and were not viewed as a substitute to care when reduced movements occurred.

The widespread availability and appeal of home Dopplers, in stark contrast with guidance against their use, represent a challenge within pregnancy care. Discussions from a priority setting partnership on women’s health technology highlighted potential tensions between the lived experience of participants who were interested in knowing how and when to use them, and midwives’ concerns about the proliferation of these devices and the challenges of discussing them^15^. Gaps remain in understanding how pregnant women navigate decisions around home Doppler use, their experiences of using the devices if they choose to do so, and how their use intersects with healthcare encounters.

The general rise in commercial products related to pregnancy and baby-care blurs the lines between medical and non-medical devices, with at-home surveillance becoming increasingly routine^16^. Bioethicists and researchers warn that at-home medical devices enter the commercial market despite a lack of evidence for benefit to health outcomes^17,18^. With options like at-home ultrasonography appearing on the horizon internationally, the scope of foetal surveillance and its impact, both on pregnant women’s lives and the foetus itself^19^, is likely to expand, making critical analysis of these technologies of the utmost importance^20^.

This study aims to explore patient and healthcare practitioner (HCP) perspectives on home Dopplers and to identify ways in which the continued availability of the devices might interface with healthcare.

## Results

Twenty interviews were conducted online or in-person with women who have experience of deciding whether or not to use a home Doppler during pregnancy (see Table 1). The sample included those who had never used a home Doppler, those who had used them in one or some pregnancies, and those who used them in all their pregnancies. Our sample included participants with a range of pregnancy and fertility experiences, including in-vitro fertilisation, miscarriage, consultant-led care for high-risk pregnancies, and stillbirth. Participant ages ranged from 25 to 43 years (mean = 33.4 years).

**Table 1:** Participant demographics – lived experience interviews

Code	Age	Ethnicity	Region	User/Non-user
LI01	35	White British	South East England	User
LI02	33	Black Caribbean	West Midlands	User
LI03	35	Asian	South East England	User
LI04	36	Mixed White and Asian	Yorkshire and the Humber	User
LI05	40	White British	North East England	User
LI06	35	White British	South West England	Non-user^a^
LI07	32	White Other	East of England	User
LI08	34	British Asian	East of England	User
LI09	35	White British	South West England	Non-user
LI10	43	White British	South West England	User
LI11	32	White British	South West England	User
LI12	38	White British	South East England	Non-user
LI13	32	White British	South East England	User
LI14	28	White British	South East England	User
LI15	30	White British	South East England	User
LI16	25	White British	South East England	User
LI17	27	White British	South East England	User
LI18	31	White British	South East England	Non-user
LI19	36	White Other	South East England	User
LI20	31	White British	South East England	User

^a^User of a mobile app that claimed to amplify the foetal heart rate, but not a handheld home Doppler.

All participants identified as women, and the sample population will therefore be referenced with female pronouns. While this paper has referred to the population of interest throughout as ‘women’, we acknowledge that transgender and non-binary individuals assigned female at birth also experience pregnancy and may undergo decision-making about home Doppler use. Further detail on recruitment and sample is detailed in the methods section. Participants’ data are presented through the categories of ‘users’ (*N* = 16) and ‘non-users’ (*N* = 4) based on the criterion of having used a handheld home Doppler device during pregnancy.

Additionally, 3 focus groups and 8 interviews were conducted with 15 self-identified HCPs (distinct from those in the patient sample with HCP backgrounds). All HCP data collection was conducted online, apart from one face-to-face interview. The sample included midwives, an obstetrician, and general practitioners (see Table 2).

**Table 2:** HCP participants by speciality

Code	Specialty	Interview or focus group
HCP01	Midwife	Focus Group
HCP02	Midwife	Focus Group
HCP03	Obstetrician	Interview
HCP04	General Practitioner	Interview
HCP05	Midwife	Interview
HCP06	Midwife	Interview
HCP07	Midwife	Focus Group
HCP08	Midwife	Focus Group
HCP09	General Practitioner	Interview
HCP10	Midwife	Focus Group
HCP11	Midwife	Focus Group
HCP12	Midwife	Focus Group
HCP13	Midwife	Interview
HCP14	General Practitioner	Interview
HCP15	General Practitioner	Interview

**Fig. 1:**
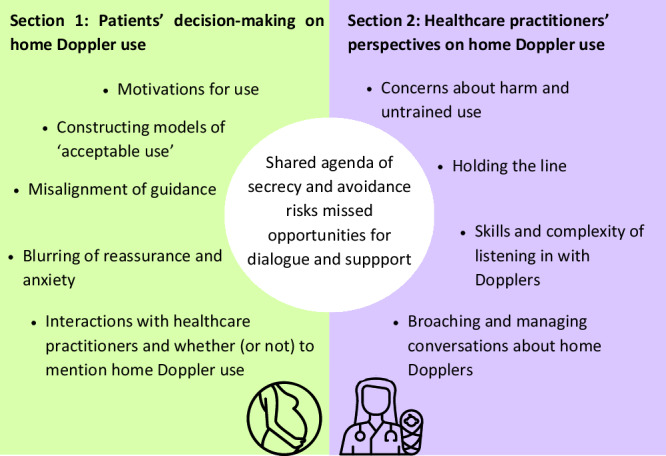
Graphic representation of thematic findings from lived-experience users and healthcare professionals, highlighting an area of overlap leading to missed conversations.

We present the findings in two sections—women’s experiences (Section 1) followed by HCP perspectives (Section 2). A visual summary of the structure and section themes is presented in Fig. 1.

### Section 1 Theme 1: Motivations for use

The primary motivations for most participants who chose to use home Dopplers were seeking early reassurance and having an opportunity to bond with the unborn baby. Use was sometimes seen as desirable as it allowed monitoring to occur in a more relaxed domestic environment compared to the clinic or hospital setting. It was also described as offering the ability to share this bonding activity with friends and family, including older siblings of the unborn baby.

I think it allowed my husband to bond a little bit antenatally, and I think it is difficult especially when dads aren’t necessarily entitled to… as much time off for antenatal care, and it allowed him to feel the excitement a bit more. -LI04, user

For some participants, usage was a means of increasing the frequency of monitoring during pregnancy. This was common before foetal movements could be felt to gauge foetal wellbeing or before the 20-week scan. Users and non-users alike could find this period an unsettling time when being able to ‘check in’ could help mitigate anxiety.

So there’s a gap between your 12-week and your 20-week scan where you have very little contact with any health services; you also have very few active symptoms of pregnancy yourself. So the first trimester’s sort of gone and so has the sickness, in my case, and then you’re also not yet feeling movement. So for me, the Doppler usage was mainly in a few weeks in there. -LI01, user

For some, self-monitoring with home Dopplers represented a means of feeling more in control. This was observed by both women with previous difficult experiences, including pregnancy loss, and also first-time pregnancies.

I just think it’s just really scary with your first pregnancy; I don’t know what a second one would be like. But I just think you don’t know what’s going on inside and you’re told of all these risks, and things that could happen, and you don’t have any control over it. -LI14, user

However, for other participants, previous difficult experiences—including high-risk pregnancies or pregnancy loss—could act as a driver against using home Doppler devices. Explanations for this included not wanting to take any risks in subsequent pregnancies and awareness that using a home Doppler may paradoxically exacerbate their anxiety about the pregnancy.

I just think that would scare me even more [to use a Doppler following previous miscarriage], I think; I’d be forever doing it sitting there trying to listen in. -LI11, user

Participants reflected on how decisions about home Doppler use occurred within a context of experiencing or perceiving that care might be difficult to access. Participants explained that although they were encouraged to contact the Maternal Assessment Unit (MAU) or call their midwife about concerns, they continued to worry that their concern may not be a priority in the context of finite NHS resources. Consequently, alternatives to self-surveillance were not always considered truly accessible.

[MAUs] are always so busy and even though they say you can come any time, it doesn’t really work that way, and sometimes it was really difficult to get through to the midwife. So yeah, here it’s a little bit like a contradictory advice they were giving, like ‘come any time’, but then you can’t really get to them. -LI19, user

For many users, accessing care and using a home Doppler were not seen as mutually exclusive. These could be held as discrete actions, with clarity that using the device was not a replacement or alternative for seeking urgent care for reduced foetal movements.

I thought, ‘If I have a lot of worries I will still go to the hospital, but I will also have the Doppler at home just for peace of mind.’-LI19, user

However, another participant spoke to a potential tension that arises when home Dopplers are available unsupervised at home, when access to care is perceived as constrained.

I can see how people are tempted to use these things as a way… as a means to like justify to themselves like, ‘I don’t need to see someone,’ or, ‘I don’t need to sit in a waiting room.’ -LI17, user

### Section 1 Theme 2: Constructing models of ‘acceptable use’

Participants, both users and non-users, developed explanatory models distinguishing ‘acceptable’ from ‘unacceptable’ uses of home Dopplers. Their accounts of decision-making revealed a moral framework differentiating ‘right’ and ‘wrong’ reasons for use. The ‘right’ reasons were generally framed as social, such as fostering emotional bonding, whereas the ‘wrong’ reasons were construed as medical, including seeking reassurance following reduced foetal movement. This moral delineation reflected participants’ awareness of stigma and anticipated judgement, contributing to the construction of home Doppler use as a taboo subject.

As long as you’re using a Doppler for, I don’t know, in inverted commas, ‘the right reasons’, whatever your particular reason is, and you’re not using it for medical reasons, or medical monitoring – I can’t really see a harm in it. -LI01, user

Users and non-users recognised the potential for home Dopplers to do harm if used in the ‘wrong’ context or in the ‘wrong’ way. A number of participants reflected that the widespread commercial availability of the devices and narratives of use amongst social and online communities was troubling and could feel predatory towards the anxiety attached to pregnancy, but that discussions around home Dopplers were hard to avoid.

I was probably quite hypocritical because I was thinking ‘these shouldn’t be on the market for anyone to buy, in the wrong hands they can be unsafe, they will potentially have problems for people who don’t escalate concerns,’ [chuckles] but the thing with that was I was still going to use it. -LI05, user

Individual participants weighed up the potential benefits and risks when deciding whether to use or not use a home Doppler. Consequently, some felt this decision was the responsibility of the pregnant woman and they should be supported to make this decision.

It’s our body, it’s our money to spend on Dopplers: help us to do it safely, don’t just say no, like we’re idiot children and it’s the 80 s and it’s OK to even talk to children like that. Yeah, so I feel very strongly that it’s a feminist issue actually and the health service needs to do better for women - listening to them, talking to them, acknowledging their rights to choose what to do with their own bodies and how to do it, and helping them to do it safely. -LI12, non-user

This feeling was strong if the participant was a healthcare professional (HCP) or in partnership with a HCP. This may have further blurred participants’ self-construction of whether their use was ‘medical’ or not, or ‘acceptable’ or not, and risked positioning official guidance as something intended for non-medical women, rather than themselves. One participant spoke of the struggle to balance the ‘mum mind’ and the ‘medical mind’ (LI15, user), which often disagreed on how much monitoring was needed.

So with being in the medical field, I knew how to monitor things and I know how to look out for things, and diagnose, so that’s really difficult to kind of accept that I’ve got to leave this alone and trust the process. -LI14, user

### Section 1 Theme 3: Misalignment of guidance

The participants we interviewed were all aware of the medical guidance against the use of home Dopplers and understood the reasons underpinning this advice. This awareness could inform their decision not to use home Dopplers, although that could still be difficult and associated with a sense of ambivalence and vulnerability:

I think even with my first pregnancy that it wasn’t really recommended to use them yourself, but I guess the temptation is always there. Yeah, and then after losing my daughter in our first pregnancy, I knew I wanted the reassurance of the Doppler… it was really hard not to go and do it. -LI09, non-user

Feeling well supported by maternity services could lessen participants’ interest in using home Dopplers. One participant experienced increased appointment frequency in her high-risk pregnancy care, and no longer felt she wanted to use the home Doppler. Other participants felt comfortable with advice to come into the MAU whenever they felt anxious rather than using a home Doppler.

They [pregnancy care team] knew how I was feeling and were sympathetic… their message that was always ‘Don’t buy it, you need to just come in and even if it is every day…’ I did just take them on their word and I then started going in more, and more, and more because that was the only way that I could feel any sort of calm about everything. -LI09, non-user

Many home Doppler users felt that absolute medical messaging did not reflect their experiences. Despite guidance to focus on baby’s movements, many used home Dopplers earlier - *before* movements were felt - as reassurance of an ongoing pregnancy.

I heard you could hear them from about… some people say eight weeks, some people say ten, so I just got one. -LI10, user

For some participants, once reassurance could be gained through feeling and monitoring foetal movements, motivation to use home Dopplers diminished, leading to reduced or discontinued use.

And then I found as I got further along in my pregnancy, after I’d have my scans I’d show and started to feel him move, I didn’t use it so much, nowhere near so much, it was just very early pregnancy mainly, and then up until about 20 weeks where I could feel the baby moving. -LI10 user

### Section 1 Theme 4: Blurring of reassurance and anxiety

Home Doppler users described how feelings of anxiety were deeply entangled with device use. For some, home Dopplers provided relief from anxiety; for others, the devices heightened it, leading to either increased or discontinued use. Some participants acknowledged that the home Doppler exacerbated their anxiety, yet found it difficult to discontinue use.

There’s like this anxiety of: ‘can I find it, and also will it be there?’ which is always a bit nerve-racking. -LI03, user

For some, experiencing additional anxiety from using a home Doppler led to decreased engagement with the device.

First I didn’t really find anything, so that escalated my anxiety, and then I thought to myself: ‘I’m not really medically trained, I don’t know what I’m looking for, and that could be a placenta flow…” I was thinking to myself: “should I be really using it?’ -LI07, user

Others continued to experience anxiety or felt that their anxiety was made worse, as the home Doppler could only offer a snapshot in time rather than continual reassurance of the unborn babies’ well-being. Concerns that the devices may not be medical-grade or appropriately calibrated were also frequently raised.

Many home Doppler users reported that they struggled to use the devices and interpret whether the output was the foetal heart rate, with this sometimes being ascribed to user error.

I didn’t know what I was doing, so I think with that it was like: ‘oh, I can’t find it, oh, I’m just not very good at it,’ rather than: ‘I can’t find it, there’s something wrong.’ -LI01, user

When a heart rate could not be confidently identified, this was typically interpreted as an issue related to device inaccuracy, user error, or foetal positioning, rather than an indicator of potential foetal problems. When a heart rate was found, there was knowledge of the potential for it to be maternal, rather than foetal.

I found it more stressful trying to put the Doppler on, like bypassing the blood flow sounds and trying to listen for a heartbeat… I think if a layperson can actually distinguish the sounds, then they’re honestly a genius. -LI08, user

A few home Doppler users expressed uncertainty about the accuracy of their home Doppler use in hindsight, wondering if they had been falsely reassured at the time.

And I still now don’t know whether I was hearing her, or whether I was hearing the placenta or anything else really, so I could have been sitting there quite happily thinking it was her and it wasn’t. -LI11, user

Participants focused on using home Dopplers to identify the presence (or absence) of a foetal heartbeat. Risks of misinterpretation and harm were conceptualised around mistaking or conflating the unborn baby’s heartbeat with their own/the maternal heartbeat.

### Section 1 Theme 5: Interactions with healthcare professionals and whether (or not) to mention home Doppler use

Most participants anticipated that HCPs would oppose home Doppler use and adjusted their interactions accordingly, often choosing not to disclose or discuss their use of the devices for fear of being judged. In a few cases, participants described their HCPs as actively instructing them not to use home Dopplers.

I had my 16-week appointment, and one of the midwives is actually my friend, but she said, ‘You’re not using one, are you?’ and I said, ‘No,’ and she said, ‘Good, we now need to ask it at every appointment that you’re not using it.’ So, it’s very much a negative sort of: ‘we’re checking in that women aren’t using them now.’ -LI11, user

Some participants chose to continue using the devices regardless of the advice.

I accepted what they were saying, but I kind of thought, ‘well, I’ll still use one anyway’ cause’ it just helps my peace of mind. – LI21, user

I didn’t tell any medical professionals what I was doing, not that I was actually hiding it, but I just wouldn’t have mentioned it: the same thing applies is because I knew there would be an element of judgement. -LI01, user

Some participants did discuss their home Doppler use with their HCPs and found them to be understanding and non-judgemental, taking the time to explain why home Dopplers should be avoided and what alternatives were available. While the women we spoke with told us that usage was consistently discouraged, some said that their HCPs seemed to recognise that use would likely continue and therefore placed emphasis on safe use, and not replacing seeking care for reduced movement.

I felt I could be completely open with them. They weren’t chastising me, they were just saying, ‘look, we completely understand why you’re using it, and these are the reasons why we’d say you shouldn’t, and certainly in X-Y-Z circumstances, never use it for reassurance, or if you do, always do it alongside, you know, seeking kind of professional medical advice’. So I felt because there was that open dialogue with them. -LI05, user

Participants proposed that HCP instruction could make home Doppler use safer, framing it as a way to bridge the gap between accessibility and perceived risk. This was based on a binary understanding of Dopplers as simply confirming or denying foetal heartbeat presence. However, participants described how this was typically met with discouragement of use overall.

So I was like, ‘Well, can you teach me how to use it please?’ and she [my midwife] was like, ‘No, I can’t.’ -LI08, user

### Section 2 Theme 1: Concerns about harm and untrained use

All of the HCPs we spoke to shared their concern about the ease of access to and untrained use of home Dopplers. Their concerns included inaccurate readings, increased maternal anxiety, and false reassurance leading to a lack of care-seeking. Some recounted personal experience of instances where false reassurance from home Dopplers had potentially contributed to missed opportunities to avoid foetal harm or death.

They also shared their uncertainty about the safety of foetal Doppler ultrasound use in pregnancy and questioned the robustness of assumptions of safety embedded in discourse and marketing, particularly with frequent use.

You see it in all aspects of baby products, don’t you, that women assume that if it’s made – it’s safe – and they assume that there is a far higher degree of regulation than there actually is… What the Doppler sound is, is an artificial recreation of a baby’s heart rate. And where’s the guarantee there that it is in any way recreating what the foetal heart rate actually is. -HCP12, midwife

Their reflections drew on professional knowledge of the skills required to use and interpret foetal Dopplers. While they noted that hearing a heartbeat was relatively simple, identifying whose heartbeat it was could be challenging. They also expressed concern that the complex expertise involved in interpreting Doppler sounds was often undervalued and overlooked.

I have third-year student midwives qualifying who, you know, are still getting to grips with it after having three years of experience, and I don’t think that that’s a reasonable thing to expect a healthcare professional to teach when it’s against the advice. -HCP05, midwife

Participants commented that when they listen in, they are not just listening for a heartbeat, but are interpreting the sound and rate within the context of other clinical information. Thus the foetal Doppler auscultation is a component of assessment, not a standalone test (as it would be used at home).

I would be explaining why we wouldn’t even listen in ourselves at 16 weeks, in line with the guidance, because of the fact that we don’t really understand as well what that foetal heart means at that stage, and the complexities of when we’re listening in to the foetal heart, and so it’s not just a number, there’s lots of things that we’re listening in to as clinicians. But yeah, it’s just people are usually quite surprised about that information. -HCP01, midwife

HCPs expressed concern that discourse (including media and device marketing) which under-played the complexity of auscultation could misrepresent the risks associated with using the devices, and potentially harm patients. Consequently, they did not advocate teaching auscultation to pregnant women as it did not capture the skill or complexity of auscultation interpretation and assessment. However, where conversations between pregnant women and HCPs did not occur, opportunities to share these explanations were missed.

### Section 2 Theme 2: Holding the line

HCP participants described pragmatically how supervised or supported home Doppler use could be an option, while recognising the associated risks and challenges in a constrained service. Crucially, in the context of clear guidance against their use, there were worries about personal and professional liability involved in engaging with conversations about Doppler devices.

[We could potentially] Just say, ‘Oh, come on, bring it in next time you see me, let’s listen together so at least I know you’re doing it in a safe way,’ ’cause you’re not gonna stop them, but you are almost… but where would we stand legally as in: ‘Well, you showed me how to use it.’ -HCP10, midwife

Balanced against these risks, this HCP expresses uncertainty about the potential for positive impact of a home Doppler. For some, this was embedded in a broader discomfort around technology superseding pregnant women’s own bodily cues.

Why do we choose Dopplers over anything else? And I think it is because we assume that technology equals safety, and yeah, regardless of whether there’s evidence for that at all, and it is effectively a crutch, or a superstition, whichever way, like these are the things that we build around ourselves to help us feel safe, whether they actually make us safer at all is a very different matter. -HCP12, midwife

Together this underpinned an approach of taking a clear line of advice to avoid using home Dopplers.

Personally, if any woman ever asks about a home Doppler, I always say no, straight out, and I just say, “No, don’t use them, because you don’t know what you’re listening to.” -HCP07, midwife

### Section 2 Theme 3: Broaching and managing conversations about home Dopplers

Conversations about home Doppler use occurred against a backdrop of explicit and well-known national guidance. This could make it difficult to engage in clinical dialogue about home Doppler use.

We kind of reiterate the national advice that it’s not recommended to use them, and I haven’t really considered the fact that we… because we don’t talk about them I suppose, it’s something that we haven’t really delved into… I don’t talk about them and I don’t recommend them. -HCP02, midwife

HCPs described tension between upholding guidance and safe practice with professionalism requiring compassion, being non-judgemental and women-centred, and creating opportunities for exploring alternative strategies or approaches through open discussion of patient concerns.

I think that’s a massive problem because patients want to make their clinicians happy, and so, even if you tell them not to do it, they’re gonna go and do it, and then if you’re doing it in an unsafe way, I’d rather you were doing it in a safe way… it’s making sure that you consult without judgement, but also maintaining your kind of professionalism in terms of what is safe to be recommended. It’s a bit like… it’s so tricky, isn’t it? -HCP04, GP

There was recognition that this culminated in home Doppler use becoming ‘a taboo topic, because the message out there is not to use them’ (HCP08, midwife), with recognition that avoidance of the topic might become a barrier to the care they were striving to deliver.

I think the minute you feel that: ‘here’s a thing that I can’t talk to them [pregnant women] about,’ you’re already just adding in a barrier there that’s not what you want when you’re kind of trying to provide care for somebody. -HCP13, midwife

Creating safe spaces in healthcare encounters for conversations about home Doppler use was therefore complex, described as “open[ing] up a big can of worms” (HCP10, midwife), and thus avoided. This occurred in a context of workload and time-pressure on appointments with competing care priorities.

Every time you ask open questions and create a discussion, it does take time, and I think that’s part of the problem is that a lot of midwives haven’t got the time to sit and have a 10-minute conversation about how somebody feels about using a home Doppler, it’s like it’s just not gonna happen. -HCP13, midwife

## Discussion

This study explored the perspectives of people with lived-experience of pregnancy and use or non-use of a home Doppler outside of medical supervision. We also spoke with HCPs who care for people during their pregnancy about their thoughts and experience with home Doppler use.

Home Doppler users primarily sought reassurance and a sense of bonding, sometimes involving partners or family, while recognising the risk of user error. Women described a complex relationship between anxiety and reassurance-seeking, with some reflecting that their home Doppler use which was intended to soothe their anxiety about the pregnancy could in fact exacerbate it. Anticipating judgement from HCPs tended to reduce discussions about Doppler use. This silence may limit opportunities for conversations about safer behaviour, alternatives for bonding, or personalised anxiety support.

HCPs expressed concerns about the availability, safety, and lack of regulation towards consumer-grade home Dopplers. Key risks identified by HCPs included inaccurate readings, increased maternal anxiety, and false reassurance leading to delays in care-seeking. Some suggested that educating pregnant individuals on proper use could mitigate risks but acknowledged the practical challenges, including limited time and resources within pregnancy care. Others resisted this approach, emphasising that foetal auscultation is a complex skill that cannot be effectively taught in a brief consultation. HCPs recognised and adhered to guidance advising against home Doppler use. Many recognised that home Doppler use remains a common practice, with this potentially going unspoken in clinical encounters. The lack of open conversations around home Dopplers risks missed opportunities to explore the underlying reasons that can motivate their use, and consider meaningful alternatives. Tackling this would necessitate a frame-shift in exploring risk and holding uncertainty, ideally in partnership.

Literature on pregnant women’s and HCPs’ views towards home Doppler use is sparse. Our study adds the previously unexplored perspectives of those who considered using the devices but chose not to, including those who have used them and then stopped in subsequent pregnancies. Through the inclusion of HCP perspectives, it also provides novel insight into how pregnant women’s experiences of home Doppler use align with and diverge from the expectations of HCPs. The study further benefits from the input of patient and public involvement panellists with relevant lived experience and the research teams’ inclusion of a range of perspectives including those of social scientists, a midwife, and a general practitioner.

While this study aimed to recruit broadly and diversely, it is unable to capture the full breadth of perspectives and experiences amongst home Doppler users, home Doppler non-users, and HCPs involved in pregnancy care. Additionally, the study design’s approach of involving self-selection (outlined below) may have produced a sample with particularly strong views on home Doppler use.

Our findings align with Middlemiss’s prior research in identifying that pregnant women’s approach to home Doppler interpretation differs from professional use. Home Doppler users in this study similarly used the devices to identify presence or absence of the foetal heartbeat, without further characterisation or contextualisation^21^. Language about ‘hearing’ the unborn baby dominated our participants’ narratives, without reported recognition or contextualisation about other aspects of home Doppler interpretation. Our findings also align with Middlemiss’s in highlighting the frequency of early use in the first trimester, with this suggesting that guidance emphasising avoiding false reassurance for reduced foetal movement may lack resonance with pregnant women^14^.

In contrast to Middlemiss’s findings, in our study, participants shared their experience of struggling to correctly or confidently identify the foetal heart rate. Several home Doppler users in this study reported being uncertain while monitoring or wondered in hindsight whether they had been falsely reassured. Further, participants demonstrated greater apprehension surrounding whether the devices were truly able to provide reassurance and relieve anxiety – with some finding that anxieties were instead amplified. Similarly to research on ultrasound scans, our findings highlight the limits of technological reassurance, as this can only provide a finite snap-shot of the unborn baby’s health^22^.

Through the integration of HCP perspectives, our study contributes a view into how anti-home Doppler guidance may inadvertently uphold a culture of silence, thus shutting down conversations that could contribute to more comprehensive care. Discussions about home Dopplers, their limitations, and risks is distinct from advocating for their use; rather, it offers a means of directly addressing the current sub-optimal landscape of opportunistic and relatively unregulated commercial markets.

Identifying the differing perceptions and ways of using Dopplers by HCPs and pregnant women has the potential to start conversations. Recognition of what home Doppler use looks like in practice—the motivations, functions, who is using them, and why—and reconciling the differences between the guiding principles of Doppler use for HCPs vs. pregnant women, is important. While they remain commercially and widely available, this potential tension remains, and this does not negate the need to ensure that commercial devices are sufficiently regulated to ensure that they do not cause harm or added risk.

Across a range of health domains, fear or anticipation of judgement from or by HCPs can act as a barrier to accessing healthcare or disclosing concerns to a clinician^23–25^. This resonates with our findings that fear of disapproval from HCPs deterred many women from talking about their use of home Dopplers. In our study, this was sometimes complicated, or justified in part, by some participants framing their home Doppler usage as social or non-medical, arguably constructing a reason as to why this was not something relevant to a healthcare setting^26^. It is noteworthy that a number of our participants were themselves medical professionals, and this can influence decisions about self-care and care-seeking, including fears about judgement and stigma. It may also have influenced their appraisal of the reported risks associated with home Doppler use, and how they pertained to them specifically^27^.

While a WHO systematic review reported that episodic diagnostic ultrasound in pregnancy is generally considered safe, this is assessed for intermittent and occasional testing and explicitly did not include continuous foetal heart rate monitoring^28^. Home Doppler devices are known to confer more risks^19,29^. Our HCP participants raised uncertainty and concerns about the safety and potential impacts of frequent (daily or more than once daily) use of home Dopplers. Without ascertaining which pregnancies this might be relevant to, it may become harder to develop observations and hypotheses that could explore and evaluate this, and this could represent crucial missing information. Potential epidemiological observational studies exploring real-world safety and impacts of home Dopplers will also be hindered by a lack of communication and therefore documentation, about their usage. It is noteworthy that Cochrane reviews exploring the evidence underpinning advice about foetal movement monitoring to characterise foetal wellbeing did not find sufficient evidence to draw conclusions to influence practice, or that antenatal CTG assessment improves perinatal outcomes^30,31^.

Anxiety is common during pregnancy, with rates in the UK reported at between 5-29.6%^32^. Anxiety occurs throughout pregnancy, for example, with a prospective study in Sweden assessing anxiety in the first trimester reporting a prevalence of 15.6%, and a study in India found that anxiety peaked during the third trimester^33^. The relationship between home Doppler use and individual level anxiety is not clear. While episodic ultrasound scans can alleviate anxiety during and after the scan, some studies report increases in anxiety before the scan^34,35^. A trial that assessed the use of home ultrasound scanning in high-risk pregnancies found this to be effective in alleviating anxiety, but this was conducted with real-time oversight and input by expert clinicians – a markedly different scenario to the usage we present here^36^. In general, research in anxiety management suggests that reassurance-seeking, whilst seemingly initially diminishing anxiety, ultimately and paradoxically increases anxiety because of a growing dependence on seeking reassurance^37^. Indeed, unravelling this is often a core component of cognitive behavioural therapy, and the cornerstone of evidence-based care in antenatal anxiety treatment. Our evidence suggests the possibility that for some pregnant women, home Doppler listening in becomes a reassurance-seeking task in the context of antenatal anxiety, and that they themselves are identifying that this is exacerbating their anxiety. If conversations about home Doppler use are not occurring, or this is not recognised as a reassurance behaviour, then this may reduce opportunities for tailored support.

There are evidence-based interventions, underpinned in the UK by national guidelines, relevant to both primary care and specialist midwifery and obstetric care which offer advice options for treatment for supporting pregnant women with impactful anxiety^38–40^. Therefore, conversations about anxiety represent potential opportunities for clinical review, individualised assessments and shared-decision making; missed opportunities for conversations represent missed opportunities to do this.

Devices that originate in medical settings and are transitioned into or become available for use at home are not new, for example home blood pressure monitors or smart watches that measure heart rhythms, and the medically supervised use of these devices is now supported by trials and research^41–43^. Home oxygen saturation probes became widely available for use, and were sanctioned and provided during the COVID-19 pandemic, although concerns remained about their accuracy or reliability in all users, for example in the presence of low oxygen saturations^44,45^. However, because their use was sanctioned, and not hidden or stigmatised, during the pandemic, the possibility of incorporating conversations about home oxygen saturation readings into clinical assessment facilitated assessment^46^. Home Doppler use has received significant concerns and is not backed by robust evidence, but nonetheless maintains widespread market availability. Given that home Dopplers are available, corporations have profited from the upholding of a lack of discussion on their use between pregnant women and HCPs. This may be doing a disservice to both HCPs and pregnant women, who need guidance and evidence that resonates with their use and the underpinning motivations, or there need to be adequate safeguards and information provision at the point of device availability.

Pregnant women and their bodies have long been understood as focal points of regulation and surveillance^47^. In a society with strong individualised notions of risk, pregnant women are compelled to engage in behaviours that mitigate current and future risks, primarily to ensure the health of their unborn baby. Monitoring and surveying the pregnant body are ways of doing this and are therefore culturally elevated as responsible behaviours. To not engage in these practices, or to engage in the wrong way, goes against moral imperatives surrounding ‘good mothers’, and is therefore stigmatised. This is illustrated through the content of advertising for baby wearable technologies, which utilise discourses of risk and responsibility in ways that may increase parental anxiety and create greater dependence on commercial products^48^.

Home Dopplers are positioned as a technology of (promised) surveillance that play into this dynamic of risk and responsibilisation during pregnancy. As we heard from our participants, they can be an attractive tool to manage anxiety, albeit sitting uneasily alongside anxiety exacerbation, and monitor a pregnancy outside of what can be, in some cases, limited clinical contact. Wang et al. highlight similar tensions in the context of the Owlet Smart Sock (a device that measures a baby’s pulse rate, oxygen levels, and sleep patterns), wherein experiences of use of devices advertised as offering ‘peace of mind’ in actuality demonstrate how usage can contribute to both the perpetuation of existing anxieties and creation of new worries^16^.

The use of home Dopplers is unsanctioned and typically constructed as unequivocally risky by the expert voices of HCPs. In this way, pregnant women are navigating a complex landscape where they may feel compelled to or have the desire to listen in, yet worry about the judgements and repercussions of being seen to engage in a practice that exposes them to risk. With an ever-growing array of technologies to engage with and monitor pregnancy, there is an increasing burden on pregnant women to navigate what is safe and what is risky^49^. In the case of a lack of discussion around home Dopplers, this confers additional responsibility onto pregnant women who may choose to use a home Doppler to do so in a way that is as safe as possible, or to explore other avenues to meet needs of bonding or reassurance around their pregnancy.

Home Dopplers remain available despite their misleading claims of providing effective monitoring and peace of mind. In the absence of an outright ban, this is likely to continue, providing uncertain benefits to pregnant women and risking active harm. This paper raises questions about how we best create space for this dialogue. While they remain available and undiscussed, we risk missed opportunities for navigating alternative routes for care and support.

## Methods

### Study design

We conducted semi-structured interviews with women with lived experience of pregnancy and who had considered using a home Doppler (*N* = 20), including both women who ultimately decided to use or not use one, to discuss decision-making around home Doppler use, experiences of using the devices (where applicable), and how these contributed to their overall experiences of pregnancy and accessing care. These were followed by interviews and focus groups with HCPs involved in routine pregnancy care (an obstetrician, midwives, and general practitioners, *N* = 15). HCP interviews and focus groups covered experiences of discussing home Dopplers with patients and reflections on the findings of the patient experience interviews.

### Recruitment and sampling

Participants with experience of home Doppler use or not were recruited through social media, regional Maternity Voices Partnership groups, and snowball sampling. Inclusion criteria were: living in England, over 18 years of age, and having prior or current experience of pregnancy which included deciding whether or not to use a home Doppler in the last 5 years. Recruitment was inclusive to those of all gender identities who met the inclusion criteria, however our sample was ultimately composed of cisgender women.

HCPs for the focus groups and interviews were recruited through professional groups, social media, and snowball sampling. Focus groups were conducted when scheduling allowed, to facilitate discussion and collective negotiation.

Patient and HCPs self-selected by contacting the research team. Upon expressing interest, patients were asked to provide their age, city of residence, and ethnicity to facilitate purposive sampling^50^. HCPs were asked to provide their role and the city where they practised.

### Data collection

Data were collected by SK, RM, and SD between August 2023 and May 2024. Interviews and focus groups were conducted over the phone, in person, or through Microsoft Teams. An overview of the sample is provided in Tables 1 and 2.

### Analysis

Coding frameworks for the patient and HCP datasets were jointly developed by SK, RM, JM, and SD through close-reading and discussion of a set of transcripts. The coding frameworks were then applied to all transcripts in NVivo 12 by SK and RM. The coding reports were consolidated into analytical themes by SK, RM, JM and SD using the One Sheet of Paper (OSOP) method^51^.

### Research team

SD and JM are clinicians (GP and midwife respectively) and all five authors work in health experiences and medical sociology. AM has experience of considering home Doppler use in pregnancy.

### Patient and public involvement

The focus of this study emerged from a priority setting partnership on technology in women’s health, wherein the safety risks associated with home Dopplers in the context of their widespread availability, were raised as a concern by midwives and women^15^. A patient and public involvement panel (PPI) was recruited through local maternity groups, personal networks, and a mailing list for patient-involvement opportunities. The panel included three members with lived experience of pregnancy who had considered whether to use a home Doppler.

The panel met to inform recruitment, sampling, topic-guide development, analysis, and dissemination. Their input ensured that our data collection and findings effectively reflected the concerns of those with lived experience of pregnancy and negotiating home Doppler use.

### Ethical approval

Ethical approval (R87247/RE001) was granted by the University of Oxford’s Central University Research Ethics Committee. We have complied with all relevant ethical regulations including the Declaration of Helsinki, including seeking informed consent from all participants.

## Data Availability

The datasets generated and/or analysed during the current study are not publicly available due to standard procedures in qualitative research regarding the privacy of interview participants. Enquiries about data can be directed towards the corresponding author.
